# Effect of chitosan and bioactive glass nanoparticles on pulp–dentin regeneration: a systematic review of *in vitro* studies

**DOI:** 10.3389/fdmed.2026.1891874

**Published:** 2026-08-26

**Authors:** Anjali Ann Cherian, Lakshmi Nidhi Rao, Aditya Shetty, Shishir Shetty, Lavanya Anumula, Krishna Prasad Shetty

**Affiliations:** 1 Nitte (Deemed to be University), AB Shetty Memorial Institute of Dental Sciences (ABSMIDS), Department of Conservative Dentistry and Endodontics. Mangalore, Karnataka, India; 2 NTR University of Health Sciences, Vijayawada, India; 3 Department of Clinical Science, College of Dentistry, Ajman University *Centre of Medical and Bio-allied Health Science Research Ajman University Al- Jruf, Ajman, United Arab Emirates

**Keywords:** bioactive glass nanoparticles, chitosan nanoparticles, pulp dentin regeneration, regenerative endodontics, reparative dentin

## Abstract

**Introduction:**

Regenerative endodontics aims to restore the structure and function of the pulp–dentin complex through biologically based therapeutic approaches. Among the various biomaterials investigated, bioactive glass (BG) nanoparticles and chitosan nanoparticles have emerged as promising candidates due to their favourable physicochemical and biological properties. BG nanoparticles can release calcium and phosphate ions that support mineralization and stimulate odontogenic differentiation. Chitosan nanoparticles have the ability to release bioactive molecules. Both have demonstrated promising biological properties associated with pulp–dentin regeneration in *in vitro* studies

**Objective:**

To systematically evaluate and critically appraise experimental *in vitro* studies investigating the effects of bioactive glass nanoparticles and chitosan nanoparticles on pulp–dentin regeneration.

**Methodology:**

A systematic literature search was conducted in PubMed, Scopus, and Web of Science following PRISMA 2020 guidelines. Experimental *in vitro* studies evaluating bioactive glass nanoparticles or chitosan nanoparticles for pulp–dentin regeneration were selected according to predefined eligibility criteria. Risk of bias was assessed using the Quality Assessment Tool for *in vitro* Studies (QUIN). Due to substantial heterogeneity in biomaterial formulations, cell types, experimental protocols, and outcome measures, the findings were synthesized qualitatively.

**Results:**

Four studies fulfilled the predefined eligibility criteria. Despite the limited number of eligible studies, the available evidence consistently demonstrated that bioactive glass nanoparticles enhanced odontogenic differentiation, mineralization, and expression of odontogenic markers in human dental pulp stem cells, whereas chitosan nanoparticle-based materials promoted cell viability, proliferation, and odontogenic differentiation under experimental laboratory conditions. However, considerable methodological heterogeneity and the exclusive inclusion of *in vitro* investigations precluded quantitative synthesis and limit the certainty of the available evidence.

**Conclusion:**

Current *in vitro* evidence suggests that bioactive glass nanoparticles and chitosan nanoparticle-based materials possess promising biological properties relevant to pulp–dentin regeneration. Nevertheless, the evidence base remains limited because of the small number of eligible studies, methodological heterogeneity, and the absence of animal and clinical investigations. Consequently, these findings should be interpreted cautiously, and well-designed standardized preclinical and clinical studies are required before the translational potential and clinical applicability of these nanoparticle-based biomaterials can be established.

**Systematic Review Registration:**

https://osf.io/upksf.

## 1 Introduction

Regenerative medicine is a field of research focused on developing living, functional tissues or organs for clinical use. In dentistry, regenerative approaches can aid in repairing and managing conditions caused by damage, absence, or loss of teeth, which may result from factors such as trauma, dental caries, periodontal disease, fractures, or genetic disorders. Within the field of regenerative medicine, regenerative endodontics focuses on biologically based strategies that facilitate the regeneration of the dentin–pulp complex by integrating stem cells, scaffolds, and bioactive signalling molecules to restore tissue structure and function (1).

When exposed to favourable conditions, pulp stem cells and other progenitor cells can differentiate into odontoblast-like cells, which contribute to the formation of reparative dentin. In contrast, reparative dentin typically exhibits a disorganized structure, which may be partially tubular and occasionally contains embedded cells. The formation of reparative dentin is generally anticipated when a bioactive material is applied directly to the exposed or amputated pulp tissue (2).

Nanoparticles have emerged as promising biomaterials in endodontics owing to their unique physicochemical properties, including a high surface area-to-volume ratio, enhanced bioactivity, antimicrobial potential, and ability to serve as carriers for therapeutic agents, making them attractive for regenerative endodontic applications. Nanoparticles, typically defined as colloidal particles measuring between 1 and 100 nm, have demonstrated promising role in various medical applications, including cancer therapy, wound healing, and endodontics (3).

Nanoparticles have emerged as multifunctional biomaterials in endodontics because they not only exhibit potent antimicrobial activity but also enhance the physicochemical properties of endodontic biomaterials, facilitate localized delivery of therapeutic agents, and promote regeneration of the dentin–pulp complex, with applications in direct pulp capping, regenerative endodontics, and root canal filling (3). Various nanoparticle systems, including silver, chitosan, bioactive glass, hydroxyapatite, calcium phosphate, mesoporous silica, zinc oxide, titanium dioxide, magnetic, and polymeric nanoparticles, have been investigated for applications ranging from root canal disinfection and biofilm control to regenerative endodontics and dentin–pulp tissue engineering (3).

Chitosan, a naturally derived biopolymer obtained from chitin, possesses intrinsic antibacterial, haemostatic, and bio adhesive properties, which contribute to its effectiveness in caries prevention, use as an intracanal medicament, and applications in regenerative endodontics (4). Transplantation of human DPSCs onto chitosan scaffolds for pulpo-dentinal complex regeneration is still in the experimental stage. Several clinical studies have reported the formation of pulp-like connective tissue with neovascularization, and in some cases, the presence of newly deposited dentin has also been observed. Chitosan nanoparticles combined with amelogenin in a hydrogel form facilitate the reorganization of calcium and phosphate ions, promoting the formation of an enamel-like structure (5).

Among the various bioactive ceramics investigated for regenerative endodontics, bioactive glass has demonstrated significant regenerative potential by promoting mineralization, odontogenic differentiation, and dentin–pulp complex regeneration. More recently, nanoscale bioactive glass has attracted interest because its higher surface area and enhanced ion release may further improve these biological responses. Furthermore, their incorporation into scaffold systems and injectable biomaterials has shown promise in improving cell attachment, proliferation, and differentiation, making them attractive candidates for next-generation regenerative endodontic therapies (1).

Although numerous studies have investigated chitosan- and bioactive glass-based materials in regenerative endodontics, the majority evaluated scaffold systems, hydrogels, composite biomaterials, or non-nanoparticulate formulations rather than isolated nanoparticles for pulp–dentin regeneration, thereby limiting their eligibility for the present review. Bioactive glass nanoparticles and chitosan nanoparticles have individually demonstrated promising biological properties, including enhanced cell viability, odontogenic differentiation, mineralization, and bioactive molecule delivery, the findings are dispersed across experimental *in vitro* studies employing diverse cell types, nanoparticle formulations, and outcome assessment methods. This methodological heterogeneity makes it difficult to draw consistent conclusions regarding their regenerative potential and limits the translation of laboratory findings into preclinical and clinical. To date, no systematic review has specifically evaluated and compared the available *in vitro* evidence on bioactive glass nanoparticles and chitosan nanoparticles for pulp–dentin regeneration. Therefore, the present systematic review was undertaken to critically synthesize the existing *in vitro* evidence, identify current knowledge gaps, and provide directions for future preclinical and clinical research in regenerative endodontics.

## 2 Methodology

### 2.1 Protocol and registration

This Systematic review was reported in accordance with the preferred reporting items for systematic reviews and Meta – Analysis (PRISMA) statement. The review protocol was registered under Open Science framework (10.17605/OSF.IO/UPKSF).

### 2.2 Protocol deviation

During the review process, the eligibility criteria were expanded to include biologically active chitosan derivatives, including chitosan monomer, because of the limited number of studies specifically evaluating chitosan nanoparticles for pulp–dentin regeneration. This modification was made to ensure a comprehensive synthesis of the available evidence and is acknowledged as a deviation from the original protocol.

### 2.3 Research question

Following the Population–Intervention–Comparator–Outcome– (PICO) framework, the research question guiding this review was: What evidence is available regarding the effects of chitosan nanoparticles or bioactive glass nanoparticles (I) on pulp-dentin regeneration (O) in *in-vitro* models relevant to pulp-dentin regeneration (P), as reported in *in vitro* experimental studies?

### 2.4 PICO framework

- P: Experimental *in vitro* models relevant to pulp–dentin regeneration, including human dental pulp stem cells (hDPSCs), rat dental pulp stem cells (rDPSCs), stem cells from human exfoliated deciduous teeth (SHED), and other dental pulp-derived cell cultures.
- I: Bioactive glass nanoparticles and/or chitosan nanoparticles used alone or incorporated into biomaterials/scaffolds for pulp–dentin regeneration.
- C: N/A
- O: Pulp–dentin regenerative outcomes, including cell viability/proliferation, cell attachment, odontogenic differentiation, mineralization, alkaline phosphatase (ALP) activity, expression of odontogenic markers (e.g., DSPP, DMP-1, RUNX2, OCN, OPN), mineralized nodule formation, and bioactivity.

### 2.5 Protocol deviation

- During the review process, the eligibility criteria were expanded to include biologically active chitosan derivatives, including chitosan monomer, because of the limited number of studies specifically evaluating chitosan nanoparticles for pulp–dentin regeneration. This modification was made to ensure a comprehensive synthesis of the available evidence and is acknowledged as a deviation from the original protocol.

### 2.6 Eligibility criteria

#### 2.6.1 Inclusion criteria

Studies were included if they fulfilled all of the following criteria:

- Original experimental *in vitro* studies investigating the regenerative effects of bioactive glass nanoparticles (BGNs), chitosan nanoparticles (CSNPs), or bioactive glass/chitosan nanoparticle-modified biomaterials specifically for pulp–dentin regeneration.
- Studies employing human or animal dental pulp-derived cells, including human dental pulp stem cells (hDPSCs), rat dental pulp stem cells (rDPSCs), stem cells from human exfoliated deciduous teeth (SHED), dental pulp cells, or other validated *in vitro* models relevant to pulp–dentin regeneration.
- Studies evaluating nanoparticles either alone or incorporated into scaffolds, hydrogels, calcium silicate cements (e.g., Biodentine), or other biomaterials, provided that the primary objective was to investigate their regenerative potential on pulp–dentin tissues.
- Because of the limited availability of studies specifically evaluating chitosan nanoparticles for pulp–dentin regeneration, studies investigating biologically active chitosan derivatives, including chitosan monomer, were also considered eligible when their primary objective was to evaluate regenerative effects on dental pulp-derived cells. Chitosan monomer is a degradation product of chitosan and retains biological activities relevant to pulp–dentin regeneration, including promotion of cell proliferation, differentiation, and mineralization.
- Studies reporting at least one biological or regenerative outcome relevant to pulp–dentin regeneration, including but not limited to:
  ○ Cell viability or cytocompatibility
  ○ Cell proliferation or adhesion
  ○ Odontogenic differentiation
  ○ Mineralization or mineralized nodule formation
  ○ Alkaline phosphatase (ALP) activity
  ○ Expression of odontogenic or osteogenic genes/proteins (e.g., DSPP, DMP-1, RUNX2, ALP, OCN, OPN, BSP, COL1A1)
  ○ Bioactivity or apatite-forming ability
- Full-text articles published in peer-reviewed journals and written in English.
- Studies providing sufficient methodological details and outcome data for qualitative synthesis.

#### 2.6.2 Exclusion criteria

The following studies were excluded:

- Review articles, systematic reviews, meta-analyses, conference abstracts, editorials, letters to the editor, case reports, book chapters, theses, or other non-original research.
- Clinical studies, case series, *ex vivo* studies, and *in vivo* animal studies without an accompanying *in vitro* experimental component relevant to pulp–dentin regeneration.
- Studies investigating bulk chitosan, microsized bioactive glass, or other biomaterials that did not incorporate chitosan nanoparticles, bioactive glass nanoparticles, or biologically active chitosan derivatives.
- Studies in which nanoparticles were used exclusively as antimicrobial agents, drug delivery vehicles, or for applications unrelated to pulp–dentin regeneration.
- Studies employing non-dental cell lines or experimental models not relevant to pulp–dentin regeneration (e.g., osteoblasts, fibroblasts, periodontal ligament cells, bone marrow-derived stem cells, or implant-related models).
- Studies that did not evaluate regenerative outcomes relevant to pulp–dentin regeneration, such as cell viability, proliferation, odontogenic differentiation, mineralization, alkaline phosphatase activity, or odontogenic gene/protein expression.
- Studies lacking sufficient methodological details or outcome data for qualitative synthesis.
- Non-English publications.
- Duplicate publications or studies reporting overlapping datasets, in which case the most complete or recent publication was included.

### 2.7 Literature search strategy

**Figure 1:**
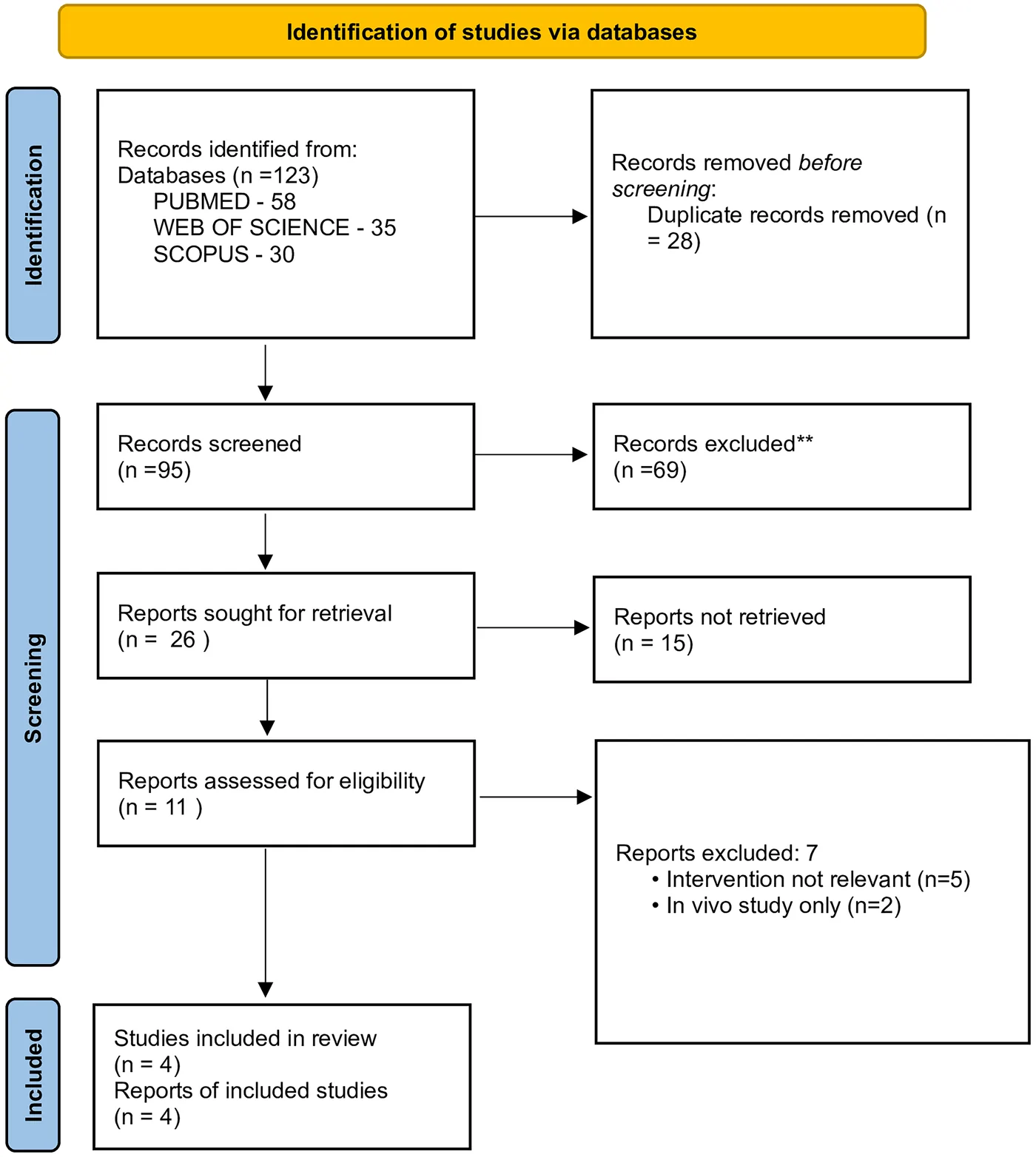
Literature search and study selection process. Flowchart illustrating the systematic literature search and study selection process. A total of 123 records were initially identified from electronic database searches conducted between 2005 and 2025 using the keywords *chitosan nanoparticles*, *bioactive glass nanoparticles*, and *pulp–dentin regeneration*. After removal of 28 duplicate records, 95 articles underwent title and abstract screening based on predefined eligibility criteria. Studies that did not meet the inclusion criteria were excluded. Full-text assessment was subsequently performed, resulting in the inclusion of four *in vitro* studies for qualitative synthesis, comprising three studies on bioactive glass nanoparticles and one study on chitosan nanoparticles.

**Figure 2:**
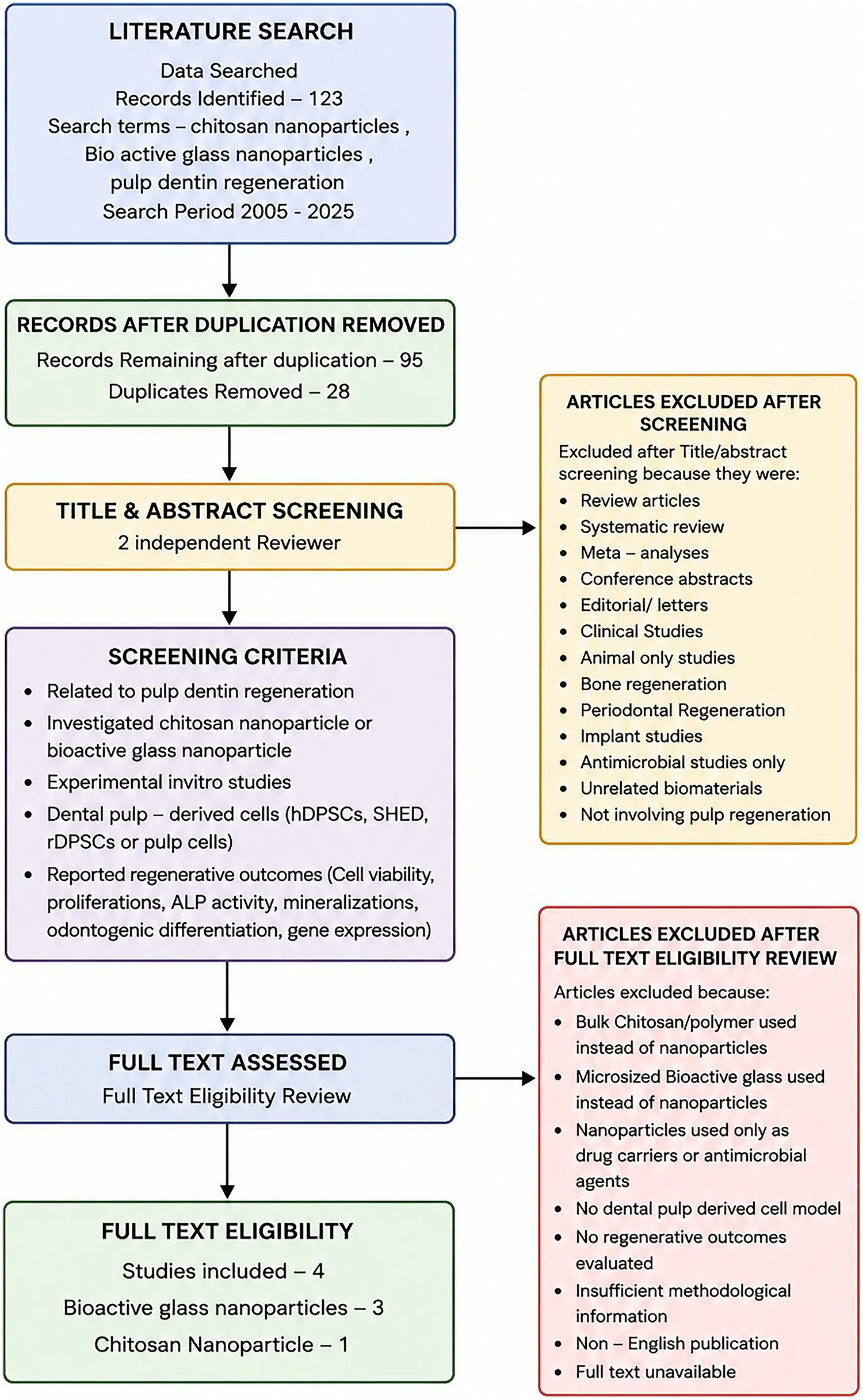
PRISMA 2020 flow diagram of the literature search, screening, eligibility assessment, and study selection process. Four *in vitro* studies met the predefined inclusion criteria and were included in the qualitative synthesis.

A comprehensive literature search was conducted in PubMed, Web of Science, and Scopus using a combination of Medical Subject Headings (MeSH) terms, free-text keywords, and Boolean operators (AND, OR) related to *chitosan nanoparticles*, *bioactive glass nanoparticles*, and *pulp–dentin regeneration*. Studies published between 2000 and 2025 were considered. All retrieved records were exported to Zotero reference management software for duplicate removal. Two reviewers independently screened titles, abstracts, and full-text articles according to the predefined eligibility criteria. The article selection process, including study identification, screening, eligibility assessment, and final inclusion, is presented in Figure 1. Any disagreements were resolved through discussion and, when necessary, in consultation with the remaining authors until consensus was achieved. During the screening process, records unrelated to pulp–dentin regeneration, non-original publications, non-English articles, and studies involving unrelated biomaterials were excluded. No automation tools were used during the study selection process. The study selection process is presented in the PRISMA 2020 diagram Figure 2 (Supplementary Table 1).

### 2.8 Data collection

Two authors independently extracted data from the included studies using the Rayyan® platform. The extracted variables included study characteristics (first author, publication year, study design, *in vitro* model/cell type, sample size, and experimental duration), details of the intervention (type of chitosan-based material or bioactive glass nanoparticle, formulation, and concentration, where applicable), outcome measures (cell viability, cell proliferation, odontogenic differentiation, mineralization, alkaline phosphatase activity, and odontogenic gene/protein expression), and the principal findings (Supplementary Table 2). Any discrepancies in data extraction were resolved through discussion until consensus was reached among the authors. The final extracted dataset was reviewed, verified, and approved by all six authors before qualitative synthesis.

## 3 Results

### 3.1 Selection of eligible studies included

The electronic literature search of PubMed, Scopus, and Web of Science identified 123 records. After removal of duplicate records based on DOI, PMID, article title, first author, publication year, and journal information, 4 unique records remained for title and abstract screening. Records were screened independently by two reviewers according to the predefined eligibility criteria. Studies unrelated to pulp–dentin regeneration, review articles, conference abstracts, editorials, clinical studies, animal-only investigations, and studies evaluating biomaterials other than chitosan nanoparticles or bioactive glass nanoparticles were excluded during this stage.

Although the initial literature search retrieved a substantial number of records, only four studies satisfied all predefined eligibility criteria and were included in the qualitative synthesis. This limited number of eligible studies primarily reflects the highly specific scope of the review rather than a lack of available literature. Most retrieved publications investigated either bulk chitosan, chitosan polymers, micro sized bioactive glass, or other nanoparticle systems, or focused on applications unrelated to pulp–dentin regeneration, such as periodontal regeneration, bone regeneration, implant coatings, antimicrobial activity, drug delivery, or restorative material development. Additionally, many studies were excluded because they were clinical or *in vivo* investigations, review articles, conference abstracts, did not employ dental pulp-derived cell models, or failed to report regenerative outcomes relevant to the review objectives. Consequently, only four *in vitro* studies fulfilled all inclusion criteria, highlighting the current paucity of experimental evidence specifically evaluating chitosan nanoparticles and bioactive glass nanoparticles for pulp–dentin regeneration.

### 3.2 Intervention characteristics of eligible studies

A total of four *in vitro* experimental studies published between 2005 and 2025 fulfilled the predefined eligibility criteria and were included in the qualitative synthesis. Of these, three studies evaluated the regenerative effects of bioactive glass nanoparticles (BGNs), either as standalone nanoparticles or incorporated into Biodentine, one study investigated the regenerative potential of chitosan monomer, a biologically active degradation product of chitosan, on dental pulp-derived cells. The included studies employed a range of dental pulp-derived cell models, including human dental pulp stem cells (hDPSCs), rat dental pulp stem cells (rDPSCs), osteoblast-like cells, and dental pulp fibroblasts, to evaluate the biological effects of these nanomaterials under controlled *in vitro* conditions.

Publication years ranged from 2005 to 2025, reflecting the gradual evolution of nanoparticle-based regenerative strategies in endodontics, with one study published in 2005, one in 2016, one in 2021, and one in 2025. Considerable methodological heterogeneity was observed among the included studies with respect to nanoparticle formulation, concentration, experimental protocols, exposure periods, and outcome assessment methods. Bioactive glass nanoparticles were evaluated at concentrations ranging from 1% to 5% (w/w) or 25–50 μg/mL, whereas chitosan was investigated at concentrations ranging from 0.0001% to 0.1%. The duration of experiments varied from 1 to 21 days, and the principal regenerative outcomes included cell viability, cell attachment, alkaline phosphatase (ALP) activity, mineralization assessed by Alizarin Red S staining, mineralized nodule formation, and expression of odontogenic and osteogenic markers. Despite methodological differences, all included studies consistently reported favourable biological responses, indicating that both bioactive glass nanoparticles and chitosan-based biomaterials enhance cellular functions associated with pulp–dentin regeneration. Owing to the substantial heterogeneity in study design, biomaterial formulations, cell models, and outcome measures, a quantitative meta-analysis was not feasible; therefore, the findings were synthesized qualitatively.

## 4 Certainity of evidence

GRADE was not applied because all included studies were *in vitro* investigations and GRADE is intended primarily for clinical evidence.

## 5 Risk of bias

The methodological quality of the included studies was evaluated using the Quality Assessment Tool for *in vitro* Studies (QUIN) (Supplementary Table 3). For studies comprising both *in vitro* and *in vivo* experimental components, only the *in vitro* component was assessed, as the QUIN tool is specifically designed to evaluate the methodological quality of *in vitro* investigations. Accordingly, for Matsunaga et al. (20), only the *in vitro* experiments were included in the risk of bias assessment.

Overall, all four studies were classified as having a moderate risk of bias, with QUIN scores ranging from 50.0% to 62.5%. The principal methodological limitations identified across the studies included the absence of sample size calculations, randomization, and blinding of outcome assessment was rarely considered. Conversely, all studies clearly stated their research objectives, incorporated appropriate control groups, implemented well-standardized experimental procedures, and employed appropriate statistical analyses. Most studies also reported clearly defined regenerative outcome measures, including alkaline phosphatase (ALP) activity, mineralization assays, and odontogenic or osteogenic gene expression. However, variations in methodological reporting were observed, particularly in Matsunaga et al. (20), which incompletely reported several methodological domains. Consequently, although the included studies provide encouraging preclinical evidence supporting the regenerative potential of bioactive glass nanoparticles and chitosan-based biomaterials for pulp–dentin regeneration, the findings should be interpreted with caution because of the overall moderate risk of bias, methodological limitations, and the inherent constraints of *in vitro* experimental research.

## 6 Discussion

The main goal of dentin regeneration is to restore its natural structure and function. For this, the formation of tubular dentin with dentinal fluid and odontoblast processes is important, as it helps in sensation, immune defence, and pulp healing (6). However, many biomolecules currently used tend to form bone-like dentin without proper tubules, which limits their ability to fully replicate the structure and function of natural dentin (7). Recent *in vitro* studies have highlighted the development of mesoporous bioactive nanoparticles, which combine small particle size with high porosity, thereby enhancing hard tissue regeneration through apatite formation mediated by calcium binding (8). In addition, spherical mesoporous bioactive nanoparticles have been reported to exhibit greater reactivity and enhanced dentin mineralization potential (9).

Lee et al. demonstrated that aminated mesoporous bioactive glass nanoparticles enhanced the odontogenic differentiation of dental pulp stem cells by increasing alkaline phosphatase (ALP) activity, mineralized nodule formation, and the expression of odontogenic markers. Similarly, Huang et al. reported that mesoporous bioactive glass nanoparticles promoted odontogenic differentiation while neutralizing the acidic microenvironment associated with pulpal inflammation. El-Fiqi et al. further demonstrated that incorporating surface-aminated mesoporous bioactive glass nanoparticles into collagen hydrogels improved scaffold stability, mechanical properties, and bioactivity, thereby providing a favourable microenvironment for hard tissue regeneration. Collectively, these findings indicate that mesoporous bioactive glass nanoparticles possess excellent biomaterial compatibility together with osteo/odontoinductive properties, supporting their potential application in pulp–dentin regeneration (10–12).

The favourable regenerative effects of bioactive glass nanoparticles observed in the included studies are supported by previous investigations demonstrating their ability to modulate the cellular microenvironment. Wu et al. showed that bioactive glass enhanced exosome production and improved their pro-angiogenic potential, whereas Hu et al. demonstrated that extracellular vesicles released from bioactive glass-containing hydrogels promoted vascularization through angiogenesis-related microRNAs. Furthermore, Shi et al. reported that bioactive glass activated vascular endothelial growth factor (VEGF)-mediated paracrine signalling, while Tian et al. demonstrated enhanced osteogenesis and angiogenesis using hierarchical porous bioactive glass. Although these studies were not performed specifically in pulp–dentin regeneration models, they provide mechanistic evidence that bioactive glass supports tissue regeneration by promoting mineralization, angiogenesis, extracellular vesicle-mediated signalling, and intercellular communication. Nevertheless, these mechanisms require validation in standardized pulp–dentin regeneration models before clinical translation can be established (13–16).

The biological properties of bioactive glass-based materials are further supported by previous reviews highlighting their regenerative potential, although the available evidence remains largely experimental (17). Findings of Nunez et al. demonstrated that incorporating nano-bioactive glass into Biodentine significantly enhanced ALP activity in human dental pulp stem cells, particularly at 7 days, suggesting accelerated odontogenic differentiation. The sustained release of calcium and silicon ions from nano-bioactive glass is believed to stimulate cell migration, adhesion, and odontogenic marker expression, thereby enhancing the biological activity of Biodentine under *in vitro* conditions (18).

Similarly, Lee et al. reported that aminated mesoporous bioactive glass nanoparticles significantly upregulated early odontogenic markers such as BSP and COL1A1, followed by increased expression of DMP1, DSPP, and OCN at later stages. Enhanced ALP activity and positive Alizarin Red S staining further confirmed increased mineralized matrix formation and odontogenic differentiation. These findings suggest that functionalized mesoporous bioactive glass nanoparticles promote dentin-forming cell differentiation under laboratory conditions, although further validation in animal and clinical studies is required (10).

Kokate et al. also demonstrated that Biodentine modified with bioactive glass nanoparticles significantly improved the biocompatibility, osteogenic differentiation, odontogenic differentiation, and mineralization potential of human dental pulp stem cells compared with conventional Biodentine. The sustained release of bioactive ions created a favourable microenvironment that supported cell proliferation, differentiation, and mineralized tissue formation, further supporting the regenerative potential of bioactive glass nanoparticle-modified biomaterials (19).

Matsunaga et al. demonstrated that chitosan promoted dental pulp tissue regeneration by enhancing cellular proliferation and differentiation (20). Chitosan was also found to increase ALP activity, suggesting stimulation of odontogenic differentiation, possibly through modulation of gene expression. In addition, bone morphogenetic protein-2 (BMP-2), an important regulator of mesenchymal progenitor cell differentiation, may contribute to this process by promoting osteogenic differentiation associated with increased ALP activity (6, 20). Furthermore, the antibacterial properties of chitosan create a favourable environment for tissue regeneration and mineralization, supporting its potential application in regenerative endodontics.

Although the overall findings were encouraging, direct comparison among the included studies was limited by considerable methodological heterogeneity. Differences in nanoparticle composition, particle functionalization, concentrations, cell sources, culture conditions, evaluation periods, and outcome assessment methods, together with the use of diverse biological assays including MTT, ALP activity, Alizarin Red staining, RT-PCR, and gene expression analyses, precluded quantitative comparison and meta-analysis. Consequently, only a qualitative synthesis of the evidence was possible. Moreover, although both chitosan- and bioactive glass-based nanoparticle materials demonstrated favourable biological properties for pulp–dentin regeneration, the available evidence is restricted to four heterogeneous *in vitro* studies, and their clinical efficacy remains unestablished. Despite the growing interest in chitosan nanoparticles, most published studies have focused on scaffold systems and antibacterial applications rather than isolated nanoparticle-based regenerative strategies, representing an important limitation of the current evidence base.

## 7 Critical appraisal

The present systematic review identified only four eligible *in vitro* studies investigating the effects of chitosan- and bioactive glass nanoparticle-based materials on pulp–dentin regeneration. Although all included studies reported favourable biological outcomes, including enhanced cell viability, odontogenic differentiation, mineralization, and expression of regenerative markers, the overall strength of the available evidence remains limited.

The included studies demonstrated considerable methodological heterogeneity with respect to nanoparticle composition, functionalization, concentrations, cell sources, culture conditions, experimental duration, and outcome assessment methods. Consequently, direct comparison between studies was difficult, and quantitative synthesis was not feasible. Furthermore, none of the studies reported sample size calculations, while randomization, and blinding of outcome assessment, were infrequently described, reducing confidence in the reproducibility of the findings. Despite these limitations, all studies employed appropriate control groups, standardized laboratory protocols, and suitable statistical analyses, supporting the internal validity of their experimental observations. A comparative summary of the mechanisms, biological effects, advantages, limitations, and overall regenerative potential of chitosan and bioactive glass nanoparticles is presented in (Supplementary Table 4).

An important limitation of the available evidence is that all included investigations were conducted under controlled laboratory conditions. Although *in vitro* models provide valuable mechanistic insights into cell behaviour and biomaterial interactions, they cannot fully replicate the complex biological environment of the pulp–dentin complex, including immune responses, vascularization, innervation, and long-term tissue healing. Therefore, the regenerative effects observed *in vitro* should not be directly extrapolated to clinical practice.

Another notable finding is the scarcity of studies specifically evaluating isolated chitosan nanoparticles for pulp–dentin regeneration. Most published literature focused on scaffold systems, hydrogels, composite biomaterials, or the antimicrobial applications of chitosan rather than nanoparticle-based regenerative approaches. This limited evidence base highlights a significant gap in the current literature and underscores the need for standardized *in vitro* investigations evaluating nanoparticle formulations specifically designed for regenerative endodontics.

Overall, the available evidence suggests that bioactive glass nanoparticles and chitosan-based nanoparticle materials possess promising biological properties relevant to pulp–dentin regeneration. However, because of the limited number of studies, moderate methodological quality, and substantial heterogeneity, the certainty of the evidence remains low. Future studies should adopt standardized experimental protocols with rigorous methodological reporting and should be followed by well-designed animal studies and clinical trials to establish the translational potential of these biomaterials.

## 8 Strengths and limitations

This systematic review has several strengths. To our knowledge, it is among the first systematic reviews to specifically synthesize the available *in vitro* evidence on the role of chitosan- and bioactive glass nanoparticle-based materials in pulp–dentin regeneration. The review followed the PRISMA 2020 guidelines, employed a predefined PICO framework, and conducted a comprehensive literature search across multiple electronic databases. A structured data extraction process and methodological quality assessment were performed to provide a transparent evaluation of the available evidence. Although only four *in vitro* studies met the eligibility criteria, limiting the breadth of the available evidence. Second, substantial heterogeneity existed among the included studies with respect to biomaterial formulations, nanoparticle concentrations, cell models, culture conditions, experimental protocols, and outcome measures, precluding quantitative synthesis and direct comparison of results. Third, all included studies were laboratory-based, and therefore the findings cannot be directly extrapolated to clinical practice. In addition, moderate methodological quality was observed across the included studies, with limited reporting of sample size justification, randomization, blinding, and operator variability. Although a comprehensive literature search was performed, most published studies investigating chitosan and bioactive glass in regenerative endodontics focused on scaffold-based systems, hydrogels, or composite biomaterials rather than evaluating chitosan or bioactive glass nanoparticles as the primary intervention. Finally, the restriction to English-language publications may have resulted in the exclusion of relevant studies published in other languages. Another limitation of this review is that grey literature sources, including conference proceedings, dissertations, preprints, and other unpublished studies, were not searched. Furthermore, manual screening of reference lists was not performed. As a result, relevant evidence may have been overlooked, potentially increasing the risk of publication bias.

## 9 Clinical implications

The findings of this systematic review suggest that chitosan- and bioactive glass nanoparticle-based materials exhibit favourable biological properties, including enhanced cell viability, odontogenic differentiation, and mineralization under *in vitro* conditions. These materials may represent promising candidates for incorporation into future pulp-capping materials, scaffolds, and regenerative endodontic biomaterials. However, the current evidence is limited to laboratory-based investigations, and therefore no recommendations for routine clinical use can be made at present. Translation of these findings into clinical practice requires validation through standardized preclinical animal studies and well-designed clinical trials to establish their safety, effectiveness, and long-term clinical performance.

## 10 Future perspective

Future research should focus on developing standardized *in vitro* protocols to facilitate meaningful comparisons between studies evaluating chitosan- and bioactive glass nanoparticle-based biomaterials for pulp–dentin regeneration. Standardization of cell sources, nanoparticle concentrations, culture duration, outcome measures, and reporting methods would improve the reproducibility and comparability of findings. Well-designed preclinical animal studies are needed to evaluate the biological performance, biocompatibility, degradation characteristics, and regenerative potential of these materials *in vivo*. Subsequently, randomized clinical trials with adequate follow-up are required to establish their safety, clinical effectiveness, and long-term outcomes. Further investigations should also explore the optimization of nanoparticle formulations, their incorporation into clinically applicable pulp-capping materials and scaffolds, and the underlying molecular mechanisms regulating odontogenic differentiation and pulp tissue regeneration.

## 11 Conclusion

Within the limitations of the available evidence, this systematic review demonstrates that chitosan nanoparticles/chitosan-based materials and bioactive glass nanoparticles exhibit promising biological properties that support pulp–dentin regeneration in *in vitro* models. Both biomaterials consistently enhanced cell viability, odontogenic differentiation, mineralization, and the expression of regeneration-associated markers, indicating their potential as bioactive materials for regenerative endodontics. However, the current evidence is limited to four heterogeneous *in vitro* studies with variations in nanoparticle formulations, experimental methodologies, and outcome assessments. Consequently, the available evidence remains insufficient to establish definitive conclusions regarding their clinical effectiveness. Standardized *in vitro* investigations, followed by well-designed preclinical and clinical studies, are required to validate these findings and facilitate the translation of nanoparticle-based regenerative strategies into clinical endodontic practice.

## Data Availability

The original contributions presented in the study are included in the article/Supplementary Material, further inquiries can be directed to the corresponding author.
