# Supplementary material for: Effect of chitosan and bioactive glass nanoparticles on pulp–dentin regeneration: a systematic review of in vitro studies
Source: Front Dent Med. 2026 Aug 26;7:1891874. doi: 10.3389/fdmed.2026.1891874 (PMC13553394; doi:10.3389/fdmed.2026.1891874)
Supplement: Supplementary file 1 [file Table1.docx]

**Table 1 Search Strategy**

| **Database** | **Search Strategy** |
| --- | --- |
| **Pubmed** | **(**  **(**  **(**  **"Chitosan"[Mesh]**  **OR chitosan[Title/Abstract]**  **OR "chitosan nanoparticle*"[Title/Abstract]**  **OR "chitosan nanoparticles"[Title/Abstract]**  **OR "chitosan nanosphere*"[Title/Abstract]**  **OR "chitosan nanocomposite*"[Title/Abstract]**  **OR "chitosan derivative*"[Title/Abstract]**  **OR "chitosan monomer"[Title/Abstract]**  **OR glucosamine[Title/Abstract]**  **)**  **OR**  **(**  **"Bioactive Glass"[Mesh]**  **OR "bioactive glass"[Title/Abstract]**  **OR "bioactive glass nanoparticle*"[Title/Abstract]**  **OR "bioactive glass nanoparticles"[Title/Abstract]**  **OR "mesoporous bioactive glass"[Title/Abstract]**  **OR "mesoporous bioactive glass nanoparticle*"[Title/Abstract]**  **OR MBN*[Title/Abstract]**  **OR nBAG[Title/Abstract]**  **OR "nanobioactive glass"[Title/Abstract]**  **)**  **)**  **AND**  **(**  **"Dental Pulp"[Mesh]**  **OR "Stem Cells"[Mesh]**  **OR "dental pulp"[Title/Abstract]**  **OR "dental pulp stem cell*"[Title/Abstract]**  **OR DPSC*[Title/Abstract]**  **OR hDPSC*[Title/Abstract]**  **OR rDPSC*[Title/Abstract]**  **OR SHED[Title/Abstract]**  **OR "stem cells from human exfoliated deciduous teeth"[Title/Abstract]**  **OR "dental pulp cell*"[Title/Abstract]**  **)**  **AND**  **(**  **"pulp regeneration"[Title/Abstract]**  **OR "pulp-dentin regeneration"[Title/Abstract]**  **OR "dentin regeneration"[Title/Abstract]**  **OR "dentine regeneration"[Title/Abstract]**  **OR "dentin-pulp regeneration"[Title/Abstract]**  **OR "odontogenic differentiation"[Title/Abstract]**  **OR odontoblast*[Title/Abstract]**  **OR mineralization[Title/Abstract]**  **OR mineralisation[Title/Abstract]**  **OR "mineralized nodule*"[Title/Abstract]**  **OR bioactivity[Title/Abstract]**  **OR ALP[Title/Abstract]**  **OR DSPP[Title/Abstract]**  **OR DMP-1[Title/Abstract]**  **OR RUNX2[Title/Abstract]**  **OR OCN[Title/Abstract]**  **OR OPN[Title/Abstract]**  **OR BSP[Title/Abstract]**  **OR COL1A1[Title/Abstract]**  **)**  **)** |
| **Web of Science** | **TS=**  **(**  **(**  **chitosan**  **OR "chitosan nanoparticle*"**  **OR "chitosan nanoparticles"**  **OR "chitosan nanosphere*"**  **OR "chitosan derivative*"**  **OR "chitosan monomer"**  **OR glucosamine**  **OR "bioactive glass"**  **OR "bioactive glass nanoparticle*"**  **OR "bioactive glass nanoparticles"**  **OR "mesoporous bioactive glass"**  **OR "mesoporous bioactive glass nanoparticle*"**  **OR MBN***  **OR nBAG**  **OR "nanobioactive glass"**  **)**  **AND**  **(**  **"dental pulp"**  **OR "dental pulp stem cell*"**  **OR DPSC***  **OR hDPSC***  **OR rDPSC***  **OR SHED**  **OR "dental pulp cell*"**  **)**  **AND**  **(**  **"pulp regeneration"**  **OR "pulp-dentin regeneration"**  **OR "dentin regeneration"**  **OR "dentine regeneration"**  **OR "odontogenic differentiation"**  **OR odontoblast***  **OR mineralization**  **OR mineralisation**  **OR "mineralized nodule*"**  **OR bioactivity**  **OR ALP**  **OR DSPP**  **OR DMP-1**  **OR RUNX2**  **OR OCN**  **OR OPN**  **OR BSP**  **OR COL1A1**  **)**  **)** |
| **Scopus** | **TITLE-ABS-KEY**  **(**  **(**  **chitosan**  **OR "chitosan nanoparticle*"**  **OR "chitosan nanoparticles"**  **OR "chitosan nanosphere*"**  **OR "chitosan derivative*"**  **OR "chitosan monomer"**  **OR glucosamine**  **OR "bioactive glass"**  **OR "bioactive glass nanoparticle*"**  **OR "bioactive glass nanoparticles"**  **OR "mesoporous bioactive glass"**  **OR "mesoporous bioactive glass nanoparticle*"**  **OR MBN***  **OR nBAG**  **OR "nanobioactive glass"**  **)**  **AND**  **(**  **"dental pulp"**  **OR "dental pulp stem cell*"**  **OR DPSC***  **OR hDPSC***  **OR rDPSC***  **OR SHED**  **OR "dental pulp cell*"**  **)**  **AND**  **(**  **"pulp regeneration"**  **OR "pulp-dentin regeneration"**  **OR "dentin regeneration"**  **OR "dentine regeneration"**  **OR "odontogenic differentiation"**  **OR odontoblast***  **OR mineralization**  **OR mineralisation**  **OR "mineralized nodule*"**  **OR bioactivity**  **OR ALP**  **OR DSPP**  **OR DMP-1**  **OR RUNX2**  **OR OCN**  **OR OPN**  **OR BSP**  **OR COL1A1**  **)**  **)** |

**Table 2: Data Extraction Table**

| Study | Model Sample/Cell Type/n) | Material (Dose) | Duration | Assay | Group | Outcome | Result | Key Findings/Conclusion |
| --- | --- | --- | --- | --- | --- | --- | --- | --- |
| Nunez et al, 2021, In vitro study ^7^ | Bioactive Glass Nanoparticle Synthesis and Nanocomposite, DPSC’s | 2 and 5% wt of BG.  Triplicates | 3, 7 and 14 days | Calorimetric ALP assay, absorbance measured using microplate reader | BG | After 14 days of culture, the nanocomposites and BD exhibited significantly higher ALP (Alkaline Phosphatase)activity compared with GIC and control group. | + effect | The BG/BD nanocomposite demonstrated a greater ability to promote the differentiation of hDPSCs into a mineralizing lineage compared to BD. |
| Lee et al, 2016, In vitro Studies^8^ | Mesoporous Bioactive Glass Nanoparticles and DPSCs | 25 – 50 μg/mL  n = 4 | 3,7, 14, 21 days | ALP activity tested | BG | High level of ALP and ARS (Alizarin Red staining)(for dentinogenic differentiation) were detected in the MBNs–NH2–treated cells | + effect | MBNs–NH2 showed remarkable bioactivity and cellular activity due to the release of various ions and their positive surface charge, making MBNs–NH2-incorporated endodontic materials valuable in pulp dentin regeneration. |
| Kokate et al, 2025, In vitro study^9^ | Biodentin and bioactive glass NP and DPSCs | 1,3,5%.  n = 3 | 1-7 days | 2% ARS, mineralized nodules visualized microscopically | BG | Alizarin red S staining showed pronounced calcium deposits in DPSCs. BD + nBAG group exhibited significantly higher mineralization than MTA and BD groups | + effect | This combination demonstrated a greater capacity to promote DPSC differentiation into a mineralizing lineage than BD alone. |
| Matsunaga et al ^10^  In Vitro study 2005 | Chitosan | 0.0001-0.1%.  n = 3 | 1,3,7 days | ALP activity assay using p-nitrophenyl phosphate substrate | Chitosan | ALP mRNA signiﬁcantly increased after 3 days of culture with chitosan monomer (ratio, 4.08) | + effect | The chitosan monomer promotes cell proliferation and differentiation in vitro even at extremely low concentrations, while optimal tissue regeneration in pulp wounds occurs in the presence of minimal initial inflammatory response. |

**Table 3 - Risk of Bias assessment of included in vitro studies using the QUIN tool**

| **Study** | **Clearly stated aims/objectives** | **Sample size calculation** | **Sampling technique** | **Control group present** | **Randomization** | **Blinding of outcome assessment** | **Standardization of procedures** | **Outcome measures clearly defined** | **Statistical analysis** | **Operator variability considered** | **Reporting of results** | **COI** | **Score** | **Overall** |
| --- | --- | --- | --- | --- | --- | --- | --- | --- | --- | --- | --- | --- | --- | --- |
| **Nunez et al** | 2 | 0 | 1 | 2 | 0 | 0 | 2 | 2 | 2 | 1 | 2 | 2 | **62.5%** | **Moderate** |
| **Matsunaga et al** | 2 | 0 | 1 | 2 | 0 | 0 | 2 | 2 | 1 | 0 | 2 | 0 | **50%** | **Moderate** |
| **Lee et al** | 2 | 0 | 1 | 2 | 0 | 0 | 2 | 2 | 2 | 1 | 2 | 2 | **62.5%** | **Moderate** |
| **Kokate et al** | 2 | 0 | 1 | 2 | 0 | 0 | 2 | 2 | 2 | 1 | 2 | 2 | **62.5%** | **Moderate** |

Score Definition

Score 0- Not reported, Score1- Partially reported, Score 2- Adequately reported

Result was divided into - low risk (≥67%), moderate risk (50%–66%), or high risk (<50%)

**Table 4 - Comparison of Chitosan and Bioactive Glass Nanoparticles for Dental Pulp Regeneration**

| **Parameter** | **Chitosan Nanoparticle** | **Bioactive Glass Nanoparticle** |
| --- | --- | --- |
| **Mechanism** \| \| | Promotes cell proliferation and osteogenic differentiation | Releases Ca/Si ions and enhances mineralization |
| **Dose range** | 0.0005–0.5% (w/v) | 1–5 wt% or up to 50 μg/mL |
| **Cell model** | Human pulp cells, osteoblast-like cells | hDPSCs |
| **Main readouts** | ALP activity, ALP expression, BMP-2 expression | ALP, ARS staining, gene expression, mineralization |
| **Advantages** | Biocompatible, biodegradable, cost-effective | High bioactivity, strong mineralization potential |
| **Limitations** | Limited direct mineralization evidence | Variable formulations and concentrations across studies |
| **Overall effect** | Enhances cellular differentiation and repair \| In vitro | Enhances odontogenic differentiation and hard tissue formation in vitro |
